# Microfluidic Enhancement of Intramedullary Pressure Increases Interstitial Fluid Flow and Inhibits Bone Loss in Hindlimb Suspended Mice

**DOI:** 10.1002/jbmr.74

**Published:** 2010-02-23

**Authors:** Ronald Y Kwon, Diana R Meays, W Joyce Tang, John A Frangos

**Affiliations:** 1 La Jolla Bioengineering InstituteLa Jolla, CA, USA; 2 Department of Biology, San Diego State UniversitySan Diego, CA, USA

**Keywords:** interstitial fluid flow, mechanotransduction, FRAP, shear stress, intramedullary pressure

## Abstract

Interstitial fluid flow (IFF) has been widely hypothesized to mediate skeletal adaptation to mechanical loading. Although a large body of in vitro evidence has demonstrated that fluid flow stimulates osteogenic and antiresorptive responses in bone cells, there is much less in vivo evidence that IFF mediates loading-induced skeletal adaptation. This is due in large part to the challenges associated with decoupling IFF from matrix strain. In this study we describe a novel microfluidic system for generating dynamic intramedullary pressure (ImP) and IFF within the femurs of alert mice. By quantifying fluorescence recovery after photobleaching (FRAP) within individual lacunae, we show that microfluidic generation of dynamic ImP significantly increases IFF within the lacunocanalicular system. In addition, we demonstrate that dynamic pressure loading of the intramedullary compartment for 3 minutes per day significantly eliminates losses in trabecular and cortical bone mineral density in hindlimb suspended mice, enhances trabecular and cortical structural integrity, and increases endosteal bone formation rate. Unlike previously developed modalities for enhancing IFF in vivo, this is the first model that allows direct and dynamic modulation of ImP and skeletal IFF within mice. Given the large number of genetic tools for manipulating the mouse genome, this model is expected to serve as a powerful investigative tool in elucidating the role of IFF in skeletal adaptation to mechanical loading and molecular mechanisms mediating this process. © 2010 American Society for Bone and Mineral Research.

## Introduction

Bone is well established to be a mechanosensitive organ that alters its structure to suit its mechanical environment. Owing to the strong anabolic potential of mechanical loading and its role as one of the primary natural factors governing bone strength,(1) the skeletal mechanotransduction pathway is recognized as a strong potential target for the development of pharmacologic and/or biomechanical therapies for bone loss. However, the development of such treatments requires a better understanding of the physical signal(s) driving loading-induced adaptation and the cellular and molecular mechanisms mediating this process.

Although the process by which mechanotransduction occurs in bone is largely unknown, a growing body of evidence suggests that adaptation to mechanical loading is mediated by skeletal interstitial fluid flow (IFF), which is altered under conditions of loading or disuse.(2) For example, deformation gradients within the tissue give rise to local pressure gradients within the matrix that drive IFF through the lacunocanalicular system, subjecting osteocytes to enhanced IFF.(2,3) If the pressure gradients are localized near the endosteal surface, mechanical loading is also likely to drive fluid flow through the endosteal surface,(4,5) exposing surface-lining cells to flow. In addition, IFF is generated through elevations in intramedullary pressure (ImP) associated with mechanical loading and/or habitual activity.(6) Increases in ImP may be induced by deformations in the matrix that result in volumetric decreases in the intramedullary cavity(6) or interactive effects between muscle activity and capillary filtration in bone tissue.(7,8) Such increases in ImP are expected to expose cells on the bone surface and within the tissue to enhanced IFF.

A large body of in vitro evidence has accumulated over the past two decades indicating that fluid flow induces a variety of responses supporting bone formation and/or inhibiting resorption in bone cells.(9–20) In contrast, there is much less in vivo evidence demonstrating that IFF mediates skeletal adaptation to mechanical loading.(21) This lack of in vivo evidence is due in large part to the technical challenges associated with decoupling IFF from matrix strain.(21) For example, although several models aimed at decoupling IFF from tissue deformation have been developed,(21–25) no model currently exists for dynamically modulating IFF in the absence of significant matrix strain within mice, which are the primary animal model for investigating the genetic contributions to skeletal mechanotransduction.(26) Indeed, the difficulties in decoupling IFF from matrix strain are exacerbated by the relatively small murine skeleton, which makes manipulation of the microscopic skeletal fluid spaces extremely challenging.

Overcoming these technical challenges in the development of a modality that decouples IFF from matrix strain within mice would advance our understanding of IFF-mediated skeletal adaptation in a number of ways. For example, such a modality would greatly facilitate the elucidation of specific molecular mechanisms activated by IFF in vivo by allowing for the investigation of the effects of altered gene expression in transgenic, total knockout, and/or conditional knockout mice. In addition, comparative analyses of the differential mechanosensitivity in inbred(27) or congenic(28) mouse strains may be used to elucidate genetic factors controlling responses to IFF. Finally, several well-established models for investigating skeletal adaptation to mechanical loading/unloading have been developed specifically for rodents(26,29) and may be used to investigate the effects of enhanced IFF against a background of altered mechanical loading. In particular, the rodent hindlimb-suspension model is one of the most well-established models of unloading-induced bone loss.(26) Thus the capacity to modulate IFF within mice would allow for the potential investigation of the protective effects of IFF in a model that mimics the loss that occurs under a variety of conditions, including paralysis, prolonged bed rest, and space flight.(30)

In this study, our first goal was to develop a microfluidic system for enhancing IFF within the mouse femur. Specifically, we sought to develop a modality for dynamic microfluidic delivery of fluid into the intramedullary compartment. Our second goal was to investigate the capacity of dynamic pressure loading of the intramedullary compartment to increase IFF within the lacunocanalicular system. This was achieved using a novel application of fluorescence recovery after photobleaching (FRAP) in which we measured fluorescence recovery after photobleaching of fluorescein within individual lacunae in the presence of pressure loading. Finally, our third goal was to investigate the potential for pressure loading, in the absence of osteogenic strains, to induce skeletal adaptation in hindlimb suspended mice.

## Materials and Methods

### Animals

Sixteen-week-old, skeletally mature C57BL/6J female mice (Jackson Laboratory, Bar Harbor, ME, USA) were used for all studies. In total, 15 mice were used: three for measurements of strain, three for FRAP experiments, three for measurements of ImP, and six for hindlimb suspension studies. Animals were maintained on a 12/12 hour light/dark cycle and had *ad libitum* access to standard laboratory rodent chow and water at all times. All mice were acclimated for at least 1 week prior to experimentation. All procedures were performed in accordance with the guidelines of the Institutional Animal Care and Use Committee (IACUC) at the La Jolla Bioengineering Institute.

### Microfluidic system and catheter implantation

**Fig. 1:**
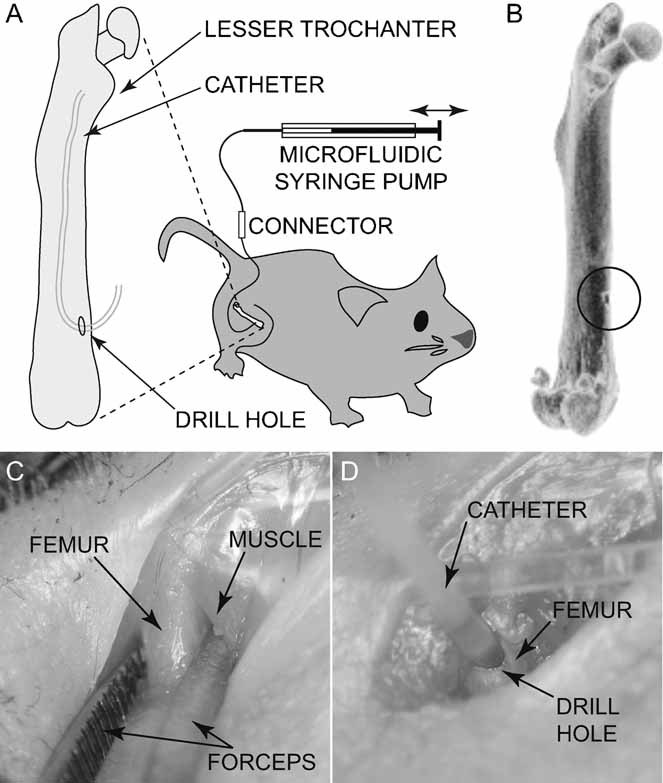
Microfluidic system for modulating ImP and IFF in alert mice. (*A*) Intramedullary pressure was enhanced by a saline-filled catheter surgically inserted into the femoral intramedullary cavity and coupled to an external microfluidic syringe pump. (*B*) µCT reconstruction of a whole femur from a cannulated mouse. In this case, the femur was harvested and the catheter removed. The drill hole where the catheter entered the intramedullary cavity is circled. A femur before and after cannulation can be seen in *C* and *D*, respectively.

Intramedullary pressure and skeletal IFF were modulated via a saline-filled catheter inserted into the femoral intramedullary cavity and coupled externally to a microfluidic syringe pump (Fig. 1*A*). For cannulation, the animals were anesthetized by intraperitoneal (i.p.) administration of ketamine (90 mg/kg) and xylazine (10 mg/kg). A small incision was made on the ventral surface of the hindlimb, and the femoral quadriceps muscles were slightly separated to expose the medial aspect of the distal femur. A small hole was drilled through the cortex into the intramedullary cavity on the medial aspect of the distal femur using a steel burr (O.D. 0.5 mm; Fine Science Tools, Heidelberg, Germany) and a high-speed microdrill (Fine Science Tools) (Fig. 1*B–D*). A saline-filled polyethylene catheter (Intramedic PE-10, OD 0.61 mm, ID 0.28 mm; Becton Dickinson, Sparks, MD, USA) was fed through the hole and routed up the intramedullary cavity such that the catheter tip was approximately 1 mm distal to the midpoint of the lesser trochanter. The hole was drilled such that the diameter was slightly smaller than the outer diameter of the catheter, resulting in a press-fit mating of the catheter with the cortical bone. The free end of the catheter was routed subcutaneously to the shoulder blades, passed through the skin (for future coupling to the syringe pump), and capped with a stainless steel tubing plug. The external catheter end was protected by outfitting the mouse with an infusion harness (Instech Solomon, Plymouth Meeting, PA, USA) and housing the free catheter end within the access port. The surgery was repeated on the contralateral limb to serve as a sham control. In this case, the free end of the catheter was tied off tightly and remained subcutaneous for the duration of the experiment. To alleviate discomfort/inflammation, analgesia was administered after surgery (meloxicam, 5 mg/kg, i.p.). To minimize potential for infection, animals received trimethoprim-sulfamethoxazole oral suspension in their water supply (0.2/0.04 mg/mL of H_2_O) for the duration of the experiment. Following surgery, all cannulated animals were ambulating normally within 24 hours. To generate dynamic ImP/IFF, the external portion of the catheter was connected to a custom syringe pump consisting of a Hamilton glass syringe mounted in a loading frame driven by a computer-controlled linear stepper motor (Anaheim Automation, Anaheim, CA, USA). For all studies, an oscillatory flow profile (∼5 Hz) with a peak pump flow rate of approximately 10 µL/s was used. This resulted in a pump stroke displacement (1 µL) that was approximately 10% of the volume of the intramedullary cavity.(21) Animals did not show a distress reaction to pressure loading, and postmortem examination 3 to 10 days following surgery revealed no leakage at the drill hole both in the absence and in the presence of pressure loading.

### Measurement of intramedullary pressure

A telemetric pressure transducer (PA-C10; Data Sciences International, St. Paul, MN, USA) was implanted to measure ImP during pressure loading, as described previously.(24,31) Following cannulation in one limb, a second hole was bored into the intramedullary cavity on the medial aspect of the femur at approximately the mid-diaphysis. The catheter tip of the transducer was inserted into the intramedullary cavity, and the transducer body was positioned subcutaneously outside the peritoneal cavity. Pressure measurements were made in the absence/presence of pressure loading using a 40 Hz lowpass filter and a sampling frequency rate of 500 Hz. For each pressure trace, the mean and peak-to-peak (2*p*_RMS_√2, where *p*_RMS_ is the root mean square of the pressure) intramedullary pressures were found following equilibration of the trace. Spectral analysis of the pressure trace was performed using a fast Fourier transform.

### Strain measurements

To measure longitudinal strain on the periosteal surface during pressure loading, femurs were immediately harvested from mice euthanized 7 days after cannulation (sodium pentobarbital, 120 mg/kg, i.p.). For each femur, the surrounding tissue was carefully removed (leaving the periosteum intact), and a single-element strain gauge (EA-06-015DJ-120; Vishay, Malvern, PA, USA) was bonded (M-Bond 200, Vishay) to the surface of the posterior femur approximately at the mid-diaphysis. Strains were recorded at 10,000 Hz using a digital acquisition system (System 6000; Vishay) in the absence and the presence of pressure loading. Spectral analysis was performed using a fast Fourier transform.

### Ex vivo measurements of lacunar FRAP

To investigate whether pressure loading enhanced cortical IFF at the lesser trochanter, we performed ex vivo measurements of fluorescence recovery after photobleaching (FRAP) of fluorescein within individual lacunae.(32,33) Seven days following cannulation in one limb, mice were administered 100 mg/kg of disodium fluorescein dissolved in PBS (tail vein). Following a 45 minute equilibration period, the mice were euthanized (sodium pentobarbital, 120 mg/kg, i.p.). The cannulated femur was quickly harvested, and tissue surrounding the femur was carefully removed, leaving the periosteum intact. The femur was clamped within a dish filled with PBS warmed to 37°C and positioned under a confocal laser scanning microscope (LSM 5 Pascal; Carl Zeiss Inc., Thornwood, NY, USA) equipped with a 40× water immersion objective (NA = 0.8) and a 14 mW Argon laser with 488 nm excitation (505 to 600 nm bandpass filter). Lacunae were imaged on the anterior aspect of the femur at the location along the femur length corresponding to the midpoint of the lesser trochanter. The image plane was adjusted such that the lacunae were within approximately 30 µm of the periosteal surface. A single lacuna was selected and photobleached by focusing the scan area on the lacuna (approximately 10× zoom) and scanning for 1 second with the acousto-optical tunable filter set to 100% transmission. Whole-field images (512 × 512 pixels) were obtained using ×1 zoom with the acousto-optical tunable filter set to 4% transmission. These images were acquired immediately before photobleaching and immediately following photobleaching for 2 minutes every 30 seconds. All images were obtained within 1 hour of the harvesting procedure to minimize effects of cell death.(32) For analysis, a region of interest outlining the photobleached lacuna was traced manually, and the mean intensity within the region was found at each time point. The logarithmic recovery ratio ln{[*I*_0_ – *I*(*t*)]/[*I*_0_ – *I*_*b*_]} was computed (Fig. 3), where *I*(*t*) is the lacunar intensity at time *t* following photobleaching, *I*_0_ is the prebleach lacunar intensity, and *I*_*b*_ is the lacunar intensity immediately following photobleaching [ie, *I*_*b*_ = *I*(0)]. The characteristic recovery time was computed as the negative inverse of the slope of the best-fit line,(33) that is, as


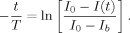


For each lacuna, three image sequences were obtained using the pressure loading scheme: no load, load, no load. The characteristic recovery time was calculated for each image sequence. The no-load recovery time was taken to be the minimum of the recovery times found from the two no-load image sequences acquired before and after pressure loading. This procedure was chosen to minimize the potential for reductions in recovery times during pressure loading arising from artifacts associated with imaging order, such as irreversible photobleaching.(33)

### Hindlimb suspension

Animals were hindlimb suspended as described previously.(24,31) Seventy two hours following cannulation, mice were hindlimb suspended for 7 days and subjected to pressure loading each day for 3 minutes per day. Sterile doses of calcein (10 mg/kg) and alizarin red (30 mg/kg) were administered subcutaneously 48 hours prior to the initiation and termination of hindlimb suspension/pressure loading, respectively. In vivo bone mineral density (BMD) measurements were obtained using a peripheral quantitative computed tomography (pQCT) scanner (XCT Research SA+, Stratec, Birkenfeld, Germany; voxel size 0.1 × 0.1 × 0.18 mm) as described previously.(24) For each animal, scans were performed 7 days prior to and at the conclusion of hindlimb suspension. For each limb, an approximately 0.36-mm-thick transverse section at the proximal region of the lesser trochanter and a 0.18-mm-thick transverse section at the mid-diaphysis were obtained for analysis. The image data were post-processed semi-automatically to determine trabecular and total BMD as described previously.(24) At the conclusion of the studies, the animals were euthanized (sodium pentobarbital, 120 mg/kg, i.p.), and the femurs were harvested, fixed in 70% ethanol, and embedded in methyl methacrylate. Embedded femurs were scanned using a micro–computed tomography (µCT) scanner (eXplore Locus, GE Healthcare, Waukesha, WI, USA; voxel size 20 × 20 × 20 µm). A 0.5-mm-thick transverse section at the proximal region of the lesser trochanter and a 0.4-mm-thick transverse section at the mid-diaphysis were used for µCT analyses. For measurements of trabecular structure, a 1-mm-diameter cylindrical region of interest within the trabecular compartment was used. The bone was segmented, and the trabecular volume fraction, thickness, and number were calculated automatically using the free image analysis program MicroView (GE Healthcare).(34) Cortical thickness at the lesser trochanter was found by manually tracing the length from the endosteal to the periosteal surface at the midpoint of the lateroanterior aspect. This aspect was chosen because it was relatively flat compared with other aspects. Cortical thickness at the mid-diaphysis was found by manually tracing the endosteal and periosteal perimeters and computing the difference of the effective periosteal and endosteal radii. For measurements of cortical thickness at the lesser trochanter and mid-diaphysis, measurements from the top, middle, and bottom image of each image stack were averaged for each transverse section. Following µCT scanning, embedded femurs were processed for analysis of fluorochrome labels by making a transverse cut at the midpoint of the lesser trochanter using a low-speed diamond-bladed saw (Buehler, Lake Bluff, IL, USA). The cut surface of the embedded bone was polished, and the surface was imaged using a confocal laser scanning microscope. Total perimeter, single label perimeter, double label perimeter, and double label area were traced manually. From these primary data, mineralizing surface per unit bone surface area (MS/BS), mineral apposition rate (MAR), and bone formation rate per unit bone surface area (BFR/BS) were calculated using standardized histomorphometric methods.(35)

### Statistical analysis

All differences between pressure-loaded bones (“load”) and sham controls (“no load”) were compared using a paired *t* test assuming unequal variances and a two-tailed distribution (*p* < .05 was considered statistically significant).

## Results

**Fig. 2:**
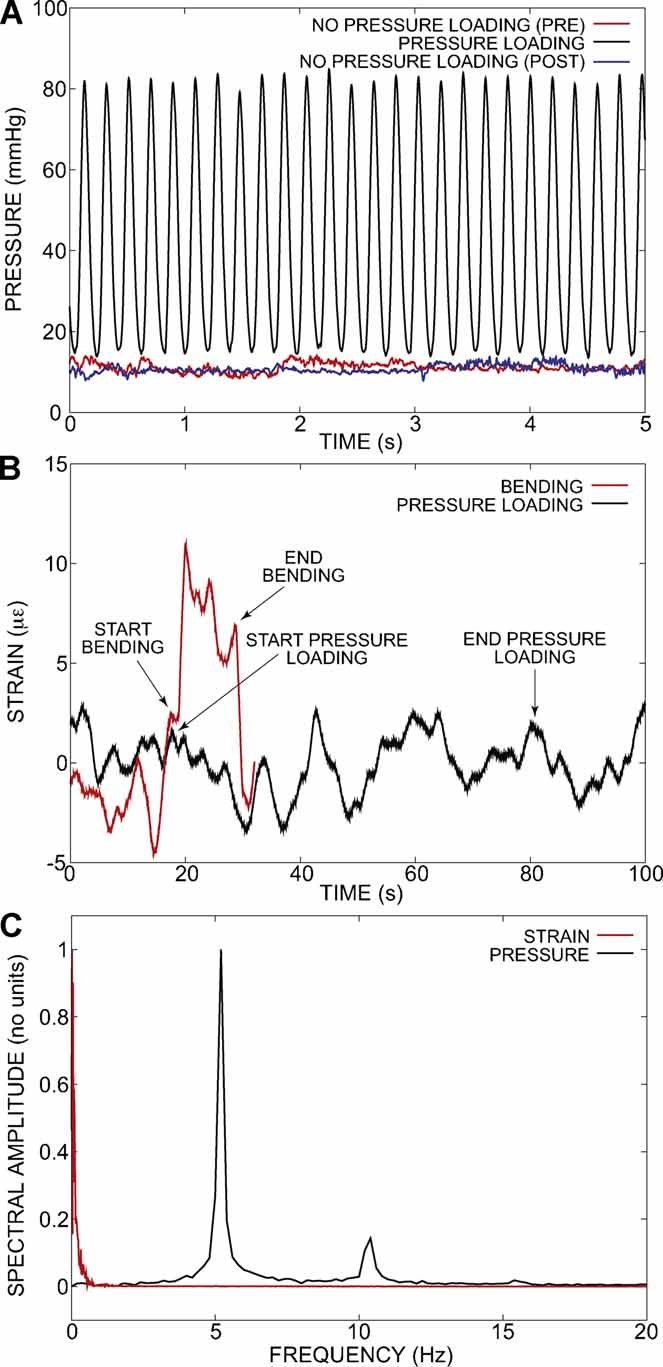
Microfluidic generation of intramedullary pressure does not induce significant tissue strain. (*A*) Representative traces of intramedullary pressure before (*red*), during (*black*), and after (*blue*) pressure loading. (*B*) Measurements of periosteal strain during pressure loading (*black*) and bending (*red*). No detectable strain was generated during pressure loading, whereas strains of approximately 10 µɛ were readily observed during bending. (*C*) Spectral analysis of intramedullary pressure (*black*) and periosteal strain (*red*) during pressure loading. A large peak at 5 Hz and smaller second harmonic peaks at 10 and 15 Hz were detected during measurements of pressure but not strain.

**Fig. 3:**
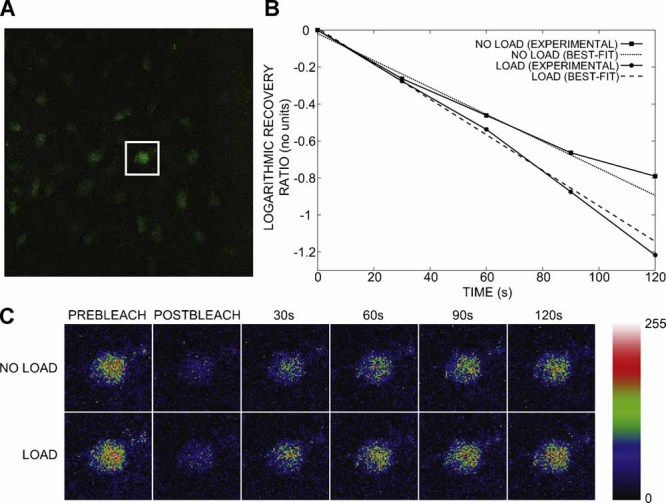
Intramedullary pressure loading enhances fluorescence recovery after photobleaching of fluorescein within periosteal lacunae. (*A*) Confocal image of fluorescein localized to lacunae. Lacunae were approximately 30 µm from the periosteal surface. (*B*) Logarithmic recovery ratios as a function of time for the lacuna circled in *A* in the absence (*solid lines with squares*) and presence (*solid line with circles*) of pressure loading in a single experiment. Linear fits of experimental data are indicated by dotted lines. Characteristic recovery times were found as the negative inverse of the best-fit lines. Pressure loading enhanced recovery, as indicated by a decreased slope during pressure loading. (*C*) Pseudo–colored image sequence of lacuna circled in *A* in the absence (*top*) and presence (*bottom*) of pressure loading. Enhanced FRAP during loading is apparent by 30 seconds.

Dynamic pressure loading increased ImP and significantly enhanced lacunocanalicular IFF in the absence of significant matrix strain. Telemetric pressure measurements revealed that pressure loading increased both mean (no load: 16.3 ± 3.6 mmHg; load: 50.3 ± 8.3 mmHg; *n* = 3) and peak-to-peak (no load: 4.1 ± 0.6 mmHg; load: 117.0 ± 67.1 mmHg; *n* = 3) ImP (Fig. 2*A*). Spectral analysis revealed a maximal peak at approximately 5 Hz (Fig. 2*C*). During ex vivo measurements of periosteal strain (*n* = 3 femurs), no increase in strain could be detected in response to pressure loading (Fig. 2*B*), and spectral analysis did not show a peak at 5 Hz (Fig. 2*C*). When we bonded (gauge side up) one of the femurs that did not exhibit detectable strain under pressure loading to an aluminum cantilever (base = 19 mm; height = 9.5 mm; *E* = 70 GPa) and applied a bending moment (0.25 N · m), we observed strains (∼10 µɛ) similar to those predicted to occur if the bending resistance of the bone was considered negligible (12.5 µɛ). This indicates that the lack of response was not due to faulty gauges and/or inadequate bonding of the gauges to the bone surface. During ex vivo measurements of FRAP, logarithmic recovery ratios decreased with time in a generally linear manner both in the presence and in the absence of pressure loading (Fig. 3). Characteristic recovery times were reduced significantly in response to pressure loading (no load: 107.7 ± 17.0 s; load: 85.5 ± 11.4 s; *n* = 8 lacunae measured from three separate experiments; *p* = .02).

**Fig. 4:**
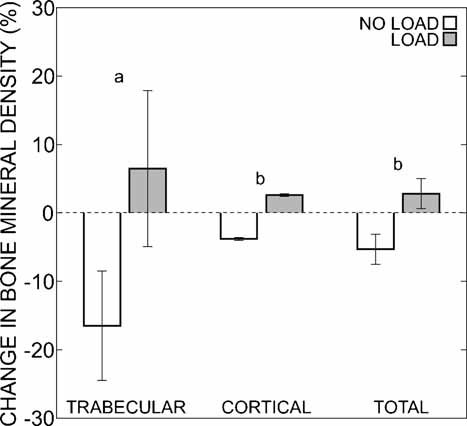
Pressure loading for 3 minutes per day significantly eliminates decreases in trabecular, cortical, and total BMD in mice subjected to hindlimb suspension for 7 days. BMD measurements were obtained in vivo via pQCT before and after hindlimb suspension. ^a^*p* < .05. ^b^*p* < .01.

**Fig. 5:**
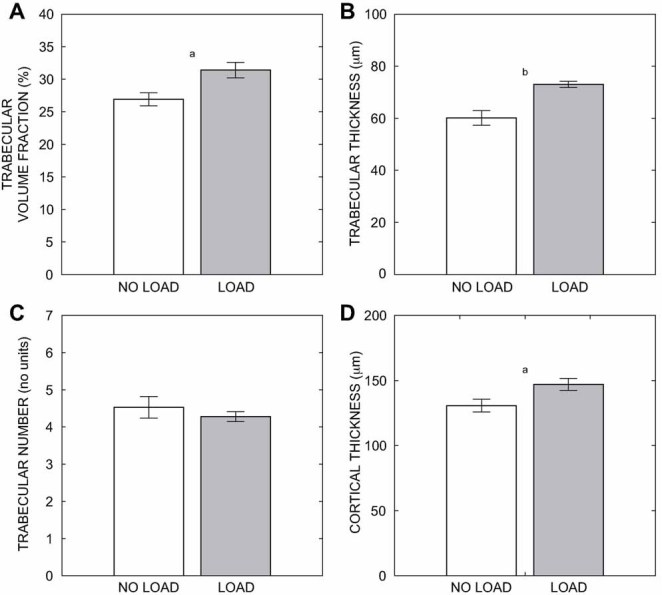
Pressure loading significantly increases trabecular bone volume fraction (*A*) and trabecular thickness (*B*) but not trabecular number (*C*). Pressure loading also increased trochanteric cortical thickness (*D*). ^a^*p* < .05. ^b^*p* < .01.

**Fig. 6:**
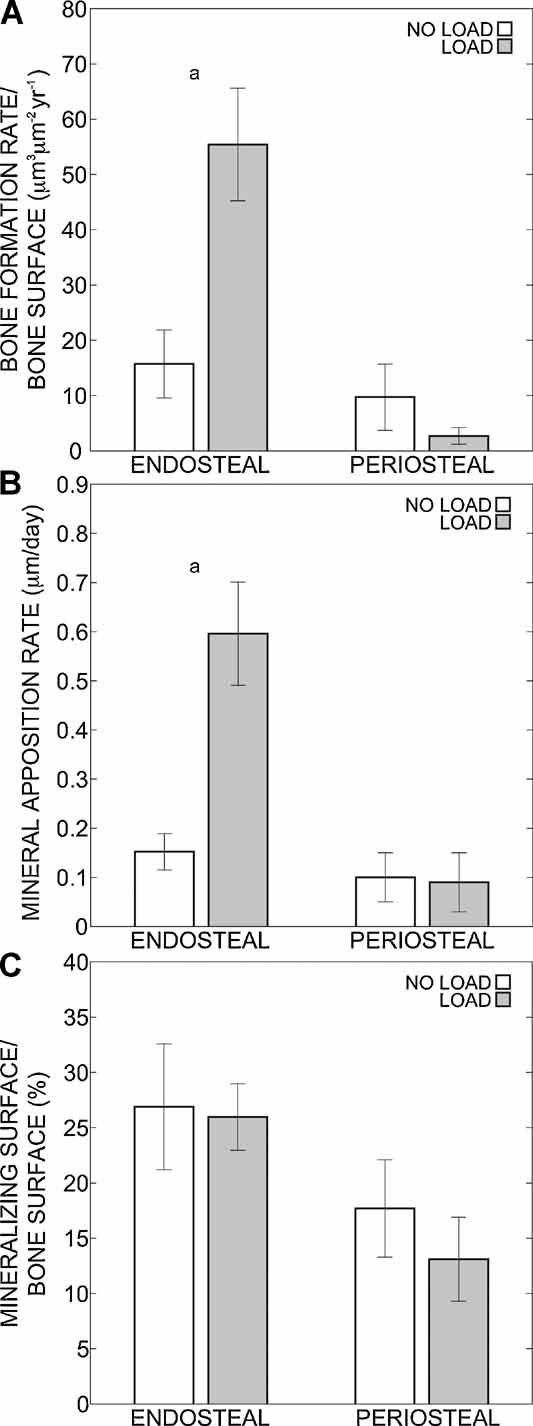
Pressure loading significantly increases bone formation rate per unit bone surface area (BFR/BS) (*A*) and mineral apposition rate (MAR) (*B*) on endosteal but not periosteal surfaces. No significant effect of pressure loading on the percentage of mineralizing surface (MS/BS) (*C*) was observed on either the endosteal or periosteal surface. ^a^*p* < .05.

In hindlimb suspended mice, pressure loading for 3 minutes per day resulted in a significant adaptive response. Decreases in trabecular BMD at the lesser trochanter were significantly eliminated by pressure loading (no load: −16.5 ± 8.0%; load: 6.5 ± 11.4%; *n* = 6; *p* = .02), as assessed by in vivo pQCT (Fig. 4). Similar trends were observed in cortical (no load: −3.8 ± 0.2%; load: 2.6 ± 0.2%; *n* = 6; *p* < .01) and total BMD (no load: −5.3 ± 2.2%; load: 2.8 ± 2.2%; *n* = 6; *p* < .01). µCT analysis at the lesser trochanter revealed that pressure loading significantly increased trabecular bone volume fraction (no load: 26.9 ± 1.0%; load: 31.4 ± 1.2%; *n* = 6; *p* = .03) and trabecular thickness (no load: 60.1 ± 2.8 µm; load: 73.0 ± 1.2 µm; *n* = 6; *p* < .01) but not trabecular number (no load: 4.5 ± 0.3; load: 4.3 ± 0.1; *n* = 6; *p* = .46) (Fig. 5*A–C*). Pressure loading also significantly increased cortical thickness at the lesser trochanter (no load: 130.7 ± 4.9 µm; load: 147.0 ± 4.6 µm; *n* = 6; p = .01) (Fig. 5*D*). Analyses of fluorochrome labels revealed differential adaptation of the endosteal (i.e., endocortical and trabecular) surface to pressure loading relative to the periosteal surface. On the endosteal surface, pressure loading increased BFR/BS significantly (no load: 15.7 ± 6.2 µm^3^/µm^2^/year; load: 55.4 ± 10.2 µm^3^/µm2/year; *n* = 6; *p* = .03) and MAR (no load: 0.15 ± 0.04 µm/day; load: 0.60 ± 0.04 µm/day, *n* = 6; *p* = .01) but not MS/BS (no load: 26.9 ± 5.7%; load: 26.0 ± 3.0%; *n* = 6; *p* = .87) (Fig. 6). On the periosteal surface, no significant differences in MS/BS (no load: 17.7 ± 4.4%; load: 13.1 ± 3.8%; *n* = 6; *p* = .47), MAR (no load: 0.10 ± 0.05 µm/day; load: 0.09 ± 0.06 µm/day; *n* = 6; *p* = .95), or BFR/BS (no load: 9.7 ± 6.0 µm^3^/µm2/year; load: 2.7 ± 1.5 µm^3^/µm^2^/year; *n* = 6; *p* = .35) were observed. At the mid-diaphysis, pressure loading resulted in a significant increase in cortical thickness (no load: 198.9 ± 3.3 µm; load: 205.2 ± 3.3 µm; *n* = 6; *p* < .01) and increased total BMD in a manner that nearly reached statistical significance (no load: 3.6 ± 1.5%; load: 9.4 ± 2.1%; *n* = 6; *p* = .056).

## Discussion

In this study we developed a novel model for the microfluidic generation of dynamic ImP/IFF within the femurs of alert mice. The model allows for the examination of both cortical and trabecular adaptation in response to dynamic ImP/IFF under conditions of normal ambulation or unloading (eg, hindlimb suspension), making it a valuable tool to investigate the anabolic/anti-resorptive potential of IFF and its role in regulating skeletal mechanotransduction. In addition, the capacity to generate dynamic ImP and skeletal IFF within mice makes it a powerful investigative tool to examine specific molecular mechanisms regulating IFF-induced adaptation, particularly given the number of modalities for manipulating the mouse genome, as well as preexisting inbred mouse strains with characterized differences in mechanosensitivity.(27)

Pressure loading significantly reduced characteristic fluorescence recovery times within individual lacunae, indicating increased solute transport owing to convection within the lacunocanalicular system.(33,36) The capacity for dynamic ImP to enhance cortical IFF is consistent with previous investigations demonstrating intramedullary pressurization-induced transcortical streaming potentials in isolated turkey ulnas(6) and increased transport of albumen in response to venous ligation–induced increases in static ImP.(31) The 26% decrease in recovery time during pressure loading was similar to the decrease observed during mechanical loading of the knee (∼24%),(33) suggesting that the levels of lacunocanalicular IFF generated by pressure loading in our study also may be achieved by mechanical loading via a modality that has been shown previously to induce a significant adaptive response.(33)

During ex vivo measurements of periosteal strain, pressurization within the skeletal interstitial fluid spaces did not result in a significant amount of tissue deformation (<10 µɛ). These findings are in agreement with theoretical treatments that have predicted tissue strains of approximately 0.1 to 1 µɛ in response to interstitial fluid pressures of 100 mmHg.(21) Importantly, these strains are far below the strains associated with habitual activity in animals and humans (∼100 to 1000 µɛ)(37) and, for similar frequencies used in our studies, much less than the strains at which skeletal adaptation occurs in mice in response to loading-induced bending (∼1000 µɛ).(38) This suggests that any adaptive responses induced by microfluidic pressure loading are attributable to effects associated with dynamic ImP/IFF rather than tissue deformation.

We found that dynamic ImP/IFF, in the presence of strains substantially less than those shown to be osteogenic, potently induces an adaptive response in trabecular and cortical bone. At the lesser trochanter, pressure loading for 3 minutes per day significantly increased BMD, trabecular volume fraction, and trabecular/cortical thickness. Pressure loading also resulted in a significant increase in cortical thickness at the mid-diaphysis, indicating that adaptation to dynamic ImP/IFF could occur relatively far from the catheter tip. Given that there were no net losses in BMD in control limbs at the mid-diaphysis, these data also indicate that adaptation could occur at a site that was relatively resistant to bone loss induced by hindlimb suspension. It is important to note that in preliminary investigations we found that cannulated animals allowed to ambulate normally showed relatively mild decreases or net increases in trabecular BMD (<10% decrease to 1% increase; data not shown), suggesting that although some losses in trochanteric BMD in hindlimb suspended mice may be attributable to effects associated with the surgical procedure, they were also attributable to hindlimb suspension.

Our data indicating a protective effect of pressure loading in hindlimb suspended mice corroborate previous findings demonstrating the capacity for dynamic intramedullary pressurization to inhibit bone loss induced by functional isolation in the turkey ulna osteotomy model(21) and suggest the potential for dynamic ImP/IFF to protect against multiple mechanisms driving unloading-induced bone loss. In particular, given the mechanical and biochemical differences that result from hindlimb suspension and functional isolation in the turkey ulna osteotomy model, it has been postulated that different mechanisms may be driving bone loss in these two models.(31) For example, while functional isolation in the turkey ulna osteotomy model has been shown to result in hypoxic osteocytes,(39,40) this has not been found to occur in hindlimb suspended mice,(31) suggesting that dynamic ImP/IFF can protect against bone loss via a mechanism that does not involve disuse-induced hypoxia. In addition, while the turkey ulna osteotomy and hindlimb suspension models both result in isolation from external mechanical loads, hindlimb suspension additionally results in a cephalic fluid shift that mimics the shift believed to be driving many of the physiologic changes that occur during space flight, including alterations in skeletal remodeling.(41) Thus these data also suggest that dynamic ImP/IFF can protect against bone loss in the presence of a cephalic fluid shift similar to the shift that occurs in microgravity.

A valuable feature of this model is that it allows for the effects of dynamic ImP/IFF on both cortical and trabecular bone to be compared within the same bone, making it ideal to investigate potential mechanistic differences in cortical and trabecular adaptation to loading-induced IFF.(42) Indeed, while the capacity for dynamic ImP/IFF to induce cortical adaptation has been demonstrated previously,(21) this study provides the first characterization of the adaptive response of trabecular bone to this stimulus. Our data indicate that trochanteric trabecular bone readily responds to pressure loading. Interestingly, there was a greater loss of trochanteric trabecular versus cortical BMD in control limbs and a greater recovery in pressure-loaded limbs, indicating a higher sensitivity of trabecular bone to hindlimb suspension and pressure loading. This may be due to the larger surface area of trabecular bone, which allows for more rapid resorption/formation. Alternatively, this difference may be due to differential sensitivity of bone cells within cortical and trabecular bone to mechanical loading-induced IFF. For example, owing to differences in local blood supply, it has been put forth that cells within individual trabeculae may have a greater reliance on transport of nutrients via loading-induced IFF relative to those within cortical bone.(42)

Histomorphometric analyses revealed that IFF-induced adaptation was confined to the endosteal surface, suggesting that this surface was exposed to higher levels of IFF and/or a biochemical environment that was more conducive to respond to pressure loading. For example, MS/BS, MAR, and BFR/BS in control limbs were lower on the periosteal surface, suggesting this surface had lower baseline osteogenic activity and thus less potential to respond to IFF. In addition, given that pressurization was achieved through a focused fluid displacement at the catheter tip, pressure loading likely generated substantial IFF within the marrow cavity tangential to the long axis of the bone, exposing endosteal cells to fluid shear stresses mirroring those shown to stimulate bone cells within parallel plate flow chambers.(9–15,43–45) Interestingly, Qin and colleagues demonstrated significant periosteal bone formation and a correlation between sites of apposition and apparent transcortical pressure gradient in pressure-loaded turkey ulnas.(21) We did not observe such a correlation (data not shown), suggesting that in generating lacunocanalicular IFF, the preferred fluid pathway was not strictly radial. Thus the capacity for dynamic pressure loading to generate IFF in a manner that correlates with transcortical pressure gradient may depend on loading profile and/or the microstructural characteristics of the skeletal fluid spaces particular to a given organism and/or anatomic site. For example, unlike mice, mature turkey bone contains osteons(46) and the interconnected fluid spaces associated with these structures,(47) which may facilitate transcortical IFF in response to intramedullary pressurization.

Our data are consistent with a role for increases in ImP/IFF arising from volumetric changes in the intramedullary cavity(6) and/or interactive effects between muscle activity and capillary filtration in bone tissue(7,8) in mediating skeletal mechanotransduction. Estimates of ImP during osteogenic mechanical stimuli in mice have yet to be attained, but the peak pressures generated in this study were of the same order of magnitude as those found previously to be generated in vitro in sheep tibia (up to 300 mmHg in response to a load of 2000 N over 0.15 second)(48) and excised human femurs (93.5 mmHg in response to a load of 980 N over 0.03 second)(49) during simulated impact. In addition, they were similar to intramedullary pressures measured in step-loaded turkey ulnas (65 mmHg)(6) in response to relatively low strains (600 µɛ). Although the peak pressures generated in this study may be considered physiologic in that they resemble those generated during impact or step compression, the ImP/IFF profiles imposed differ greatly from those expected to occur during ambulation both in magnitude and in duration. Thus it is difficult to assess whether decreased IFF was responsible for the reduction in BMD that occurred in control limbs during hindlimb suspension. For example, although the mice were exposed to pressure loading for only 3 minutes per day (as opposed to the continuous exposure of elevated ImP/IFF during ambulation), the peak pressures were much higher than the reduction in ImP (3 mmHg)(31) that occurs during hindlimb suspension.

It has been widely hypothesized that skeletal adaptation to mechanical loading is mediated by bone cell mechanotransduction of interstitial fluid shear stresses.(37,50,51) Thus it is useful to obtain an order-of-magnitude estimate for the shear stresses generated during pressure loading by approximating the lacunocanalicular IFF velocities driving observed changes in FRAP. Zhou and colleagues recently demonstrated that the capacity for dynamic IFF to enhance lacunar FRAP is governed by displacement of the solute during oscillatory flow and whether this displacement equals or exceeds the canalicular length between neighboring lacunae.(36) This implies that in our studies, for a decrease in FRAP to be observed during pressure loading, the unbleached solute likely traveled at least the entire canalicular length between neighboring lacunae (∼10 µm)(32) every 0.1 second (assuming a period of 0.2 second for a full oscillatory cycle). In this case, we estimate a lower bound for the canalicular IFF velocities of approximately 10 µm/0.1 s = 100 µm/s. By approximating the flow profile through a single canaliculus as Stokes flow through an annulus(50) and assuming a fluid viscosity of 0.001 Pa · s, a canalicular diameter of approximately 300 nm,(52) and an osteocyte process diameter of approximately 100 nm,(52) we estimate that the canalicular shear stresses were, at a minimum, on the order of approximately 1 Pa. This analysis suggests that an adaptive response, in the absence of osteogenic strains, occurred in the presence of interstitial fluid shear stresses that have been widely demonstrated to induce osteogenic and antiresorptive responses in bone cells in vitro(11,14–16) and which have been predicted to occur under habitual loading.(37)

One question regarding our studies is whether pressure rather than interstitial fluid flow may have been the physical signal driving skeletal adaptation. In vitro, pressure has been demonstrated to promote bone formation and inhibit resorption through regulating osteoblasts,(53–55) osteocytes,(56) and marrow cells.(57,58) However, it is important to note that these studies were conducted using significantly greater pressures (approximately three- to several hundred–fold greater) or, in the case of Roelofsen and colleagues,(55) much longer loading durations (twenty-fold greater) than those used in our studies. Importantly, in vitro studies investigating the dependence of peak pressure magnitude and loading duration on the capacity of bone cells to respond to pressure suggest that they may be unable to respond to the pressure profile used in this study (ie, peak pressures of approximately 100 mmHg for 3 min/day). For example, increases in intracellular Ca^2+^ concentration after approximately 1 minute of cyclic pressurization was shown to occur in bone cells exposed to 258 mmHg but not 129 mmHg.(59) In addition, cyclic pressurization with a peak pressure of 300 mmHg was found to affect osteoblast proliferation after loading for 1 hour per day but not 20 minutes per day.(54) Taken together, these data suggest that dynamic IFF rather than pressure was the primary factor driving skeletal adaptation in our studies.

Several important limitations need to be considered when interpreting our findings. First, measurements of lacunar FRAP were obtained ex vivo in harvested femurs, and it is unclear whether the pathways for IFF in response to pressure loading differ substantially ex vivo compared to those in vivo. However, it is important to note that several precautions were taken to minimize the effect of the harvesting procedure on IFF, including performing all imaging studies within 1 hour of animal sacrifice (to minimize cell death) and leaving the periosteum intact. Second, it is possible that cannulation resulted in a significant inflammatory response, particularly given that the catheter was relatively near or at the sites of analysis. It is unknown whether this inflammatory response was necessary for ImP/IFF-induced adaptation in pressure-loaded limbs to occur or enhanced this process. Inflammatory responses associated with cannulation are an inherent limitation in our study, but the confounding effects of inflammation were controlled in part by comparing pressure-loaded limbs with sham-operated controls.

In summary, we have developed a novel model for the microfluidic generation of dynamic ImP/IFF within the femurs of alert mice. Ex vivo FRAP investigations revealed that pressure loading of the intramedullary compartment significantly enhanced IFF within the lacunocanalicular system. In addition, pressure loading for 3 minutes per day eliminated losses in BMD in hindlimb-suspended mice, significantly increased indices of cortical and trabecular structure, and increased the rate of endosteal bone formation. This model provides a unique capability to dynamically and directly modulate IFF in alert mice in the absence of significant tissue strain. By allowing the investigation of skeletal adaptation to pressure loading in transgenic or knockout mice, it is expected that this model will greatly facilitate the elucidation of specific cellular and molecular mechanisms regulating adaptation to skeletal fluid flow in vivo.
